# Permeability of New Antifungal Fluconazole Derivatives through a Lipophilic Membrane: Experiment and Modeling

**DOI:** 10.3390/molecules28010389

**Published:** 2023-01-02

**Authors:** Tatyana V. Volkova, German L. Perlovich

**Affiliations:** G.A. Krestov Institute of Solution Chemistry RAS, 153045 Ivanovo, Russia

**Keywords:** fluconazole derivatives, permeability, lipophilic membrane, PermeaPad barrier, structure–permeability relationship

## Abstract

Relationships between the structures of molecules and their properties form the basis of modern chemistry and lay the foundation for structure-based drug design. Being the main two determinants of bioavailability, solubility and permeability of drugs are widely investigated experimentally and predicted from physicochemical parameters and structural descriptors. In the present study, we measure the passive diffusion permeability of a series of new fluconazole derivatives with triazole and thiazolo-pyrimidine moieties connected by different linker bridges through the PermeaPad barrier—a relatively new biomimetic lipophilic membrane that has been increasingly used in recent years. The permeability coefficients of new derivatives are shown to be dependent both on the structure of the linker fragment and on the substituent in the phenyl ring of the thiazolo-pyrimidine moiety. The impact of the compound ionization state on the permeability is revealed. Reliable correlations of the permeability with the antifungal activity and distribution coefficient are found. In addition, the solubility–diffusion approach is shown to be able to successfully predict the permeability of the studied derivatives. The obtained results can be considered another step in the development of permeability databases and design of schemes for in vitro permeability prediction.

## 1. Introduction

The design of new drug entities is an ongoing process. Among various groups of drug compounds, the antifungal class is of the greatest importance since fungal infections are widely spread throughout the world, especially in poorly developed countries. Patients with coronavirus, weak immunity and oncology are in the high-risk group, for which fungal infections are crucial and ominous [1]. The marketed antifungal derivatives of the triazole class are highly demanded by patients, e.g., fluconazole, voriconazole, terconazole, tioconazole, posaconazole, etc. [2]. The well-known representative of this class—fluconazole—serves as the basis for many newly synthesized biologically active compounds. On the other hand, the task of evaluating not only the bioactivity but also the pharmacologically relevant physicochemical properties of the new compounds is another step forward in promoting these substances to the pharmaceutical market. Among these properties, solubility in pharmacologically relevant solvents and permeability through biological membranes are of primary importance [3]. The distribution coefficient in the 1-octanol/water system (logD/logP) is another important parameter often used to predict the solubility and permeability of a substance. Since solubility and distribution coefficients of compounds with close permeability coefficients may differ by several orders of magnitude (in other words, the ranges of permeability variation are much narrower than those of solubility and distribution) [4], the task of constructing models to predict permeability seems to be more difficult than that for solubility and distribution prediction, especially in a series of structural homologs. As early as 1996, Camenisch et al. [5] revealed that permeability through a simple membrane depends on the partition coefficient as a measure of lipophilicity. However, this dependence can have a linear, bilinear (reaching a plateau or having a local maximum), sigmoid or parabolic shape. Moreover, specific dependence is not always observed. This can be due to different factors influencing the permeability. For example, polarity was confirmed to be a crucial descriptor for human intestinal absorption, permeability and blood–brain barrier penetration, especially if the compound has several polar atoms in the molecule [6]. Moreover, in the case of biological barriers and biomimetic membranes, permeation appears to be a complicated process implying several steps: desolvation, interactions with phospholipid head groups and access to the hydrophobic membrane interior [7]. Thus, the medium pH, which determines not only the ionization state of the compound (anionic, cationic, zwitterionic or neutral) but also the charge of the components of the lipophilic membrane, comes to the fore. For example, Sugano et al. [8] found the permeability of basic compounds to be higher than the value found from the 1-octanol/water partition. In addition, in our previous study of the permeability of various drugs (we studied clonixin and dofetilide as an example) through the liposome-based membrane—PermeaPad barrier—at different pHs [9], we proved that the negative surface potential of the membrane at pH 7.4 (the anionic particles of the membrane fatty acids) increases the probability of permeation of cationic species and slows down the anion movement.

Regardless, or perhaps because, of the complexity of the task, the search for prediction schemes for the permeability of drugs, especially newly synthesized pharmaceutical entities, has, for many decades, attracted the constant attention of the medicinal scientific community. Approaches based on quantitative structure–property relationships (QSPRs) serve as a convenient tool for selecting synthesis objects with desired pharmacological properties. As a result, the correlation dependences between the permeability and available descriptors of compounds, including their structural analogues, not only allow predictive models to be built, but also make it possible to better understand the factors influencing their permeability. At the same time, dealing with structurally related substances increases the probability of reliable correlations.

**Figure 1:**
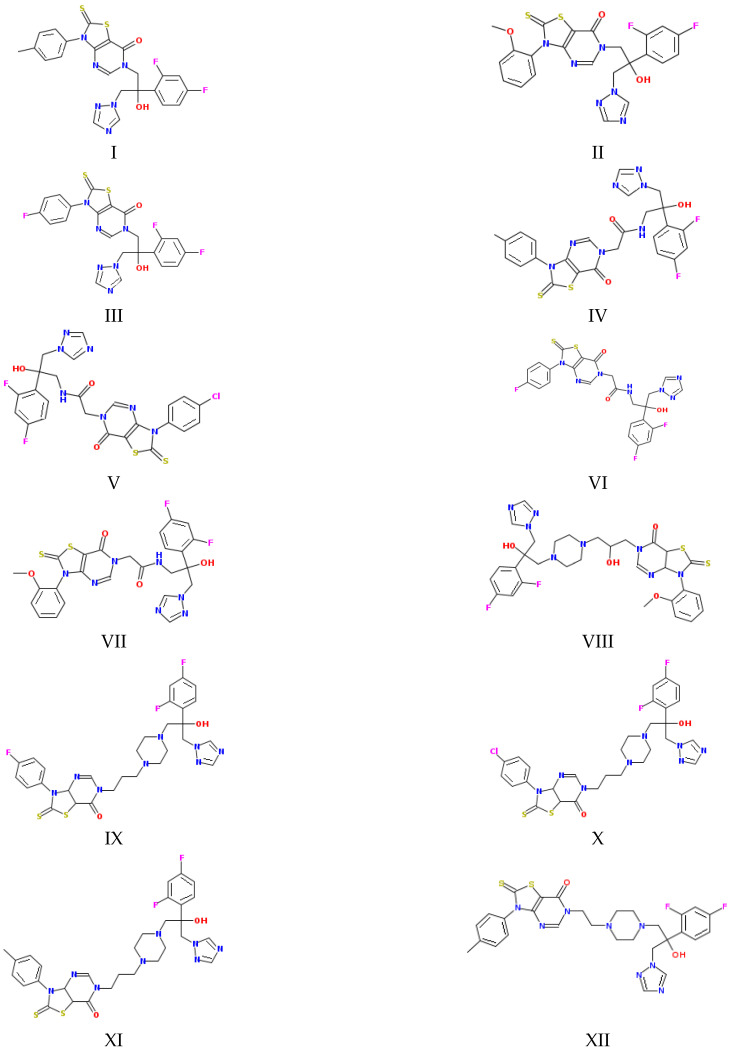
Structures of the studied compounds (see the names in Section 2.1).

Taking into account the numerous attempts to predict permeability reported in the literature [10,11,12] and our own experience [9,13,14,15], in the present study, we aimed to find a correlation between the permeability of a number of new fluconazole antifungal derivatives (Figure 1), measured using the lipophilic PermeaPad barrier and several physicochemical properties and descriptors. In addition, we derived a reliable correlation equation for permeability prediction in the framework of the solubility–diffusion theory using the calculated diffusion coefficients in water and 1-octanol and the distribution coefficient in the 1-octanol/buffer pH 7.4 system determined experimentally in the literature [16]. Notably, this approach seems, to us, quite promising, although, to the best of our knowledge, it has been rarely used by other researchers. In addition, taking into account the significance of the interconnection between biological activity and physicochemical properties, we determined the relationship between the permeability and antifungal activity.

## 2. Results and Discussion

### 2.1. Coefficients of Permeability through the PermeaPad Barrier

**Figure 2:**
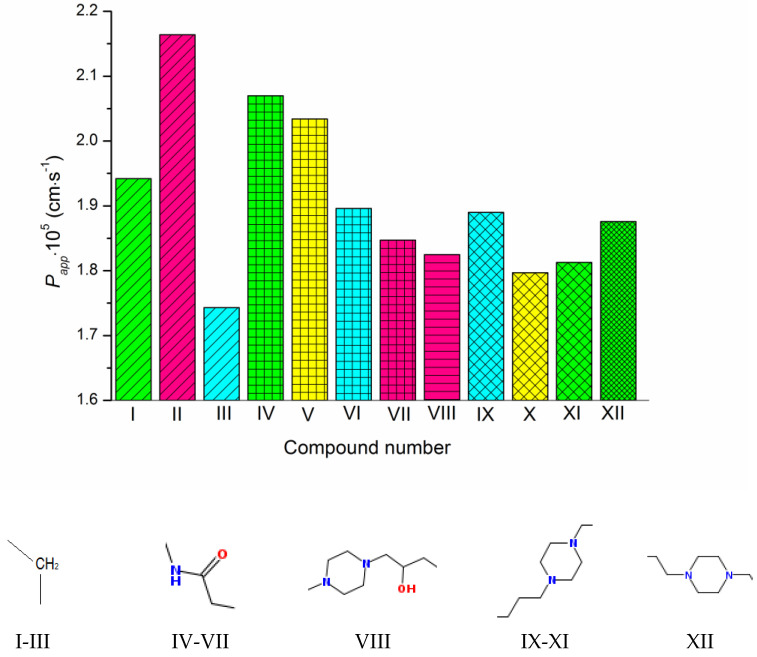
Experimental permeability coefficients of the studied compounds. The structures of the linkers between the triazole and pyrimidino[4,5-d]thiazol heterocycles are shown under the diagram. The columns referring to the same linkers are shaded in the same way. The substituents at the phenyl ring are colored as follows: CH3—green, OCH3—pink, F—cyan, Cl—yellow. The compound numbering can be found in Figure 1.

The permeability coefficients of the studied derivatives of fluconazole measured experimentally are listed in Table 1 and Figure 2. Table S1 represents the concentrations of the donor solutions and steady-state fluxes through the PermeaPad barrier. In addition, Table 1 contains the diffusion coefficients of the compounds calculated by Equation (10) taking into account the hydrodynamic radii of the solutes, the dynamic viscosities of the solvents (water or 1-octanol) and the distribution coefficients in the 1-octanol/buffer pH 7.4 system taken from the literature [16]. Expectedly, the diffusion coefficients in 1-octanol are considerably lower than in water (7–8-fold). Moreover, the diffusion slows down in both solvents with molecular-weight growth.

As Figure 1 and Figure 2 show, the molecules of the substances are composed of triazole and thiazolo-pyrimidine moieties connected by different linkers, illustrated in Figure 2 above the columns in the following order: methylene (I–III), acetamide (IV–VII), hydroxypropylpiperazine (VIII), propylpiperazine (IX–XI) and ethylpiperazine (XII). In addition, four kinds of substituents at the benzene ring are used: CH_3_, OCH_3_, F and Cl. First of all, we tried to trace the influence of the structural features on the apparent permeability. According to Figure 2, the permeation rate is influenced by both the linker structure and the substituent nature. In the group of compounds with the methylene linker, we determined the maximal (for methoxy-substituted) and the minimal (for fluoro-substituted) permeability, indicating the most pronounced effect of the substituent on the permeability within this group. The permeability within the group with the acetamide bridge fragment decreased in the following order: methyl- (CH_3_) > chlorine- (Cl) > fluoro- (F) > methoxy- (OCH_3_). Interestingly, the permeability values of the methyl-substituted substances with acetamide (VII) and hydroxypropylpiperazine (VIII) linkers were very close and, at the same time, were essentially lower than that of the methyl-substituted compound (II) from the group with the methylene bridge. Probably, the presence of the acceptor nitrogen atom had a negative effect on the molecule permeability. In addition, the lower molecular weight (see Table S2) of the compound (II) molecule as compared to (VII) and (VIII) could also be responsible for the permeability reduction. A comparison of methyl-substituted compounds (XI) and (XII) shows that the elongation of the alkyl chain of the linker fragment lowers the permeability. Expectedly, the shortening of the alkyl chain combined with the exclusion of the piperazine ring from the linker moiety (compound I) improves the permeability in the group of methyl-substituted derivatives. Interestingly, the permeability decrease was detected for the methoxy-(OCH_3_)-substituted substances: I (methylene linker) < VII (acetamide) < VIII (hydroxypropylpiperazine), which agrees with the order in which the molecular weight decreased as the bridge fragment connecting the triazole and thiazolo-pyrimidine moieties became bigger. The permeability of the Cl-substituted compounds P_app_ (V) > P_app_ (X) was in complete agreement with their molecular weights, as the linker structural unit grew bigger in size.

**Figure 3:**
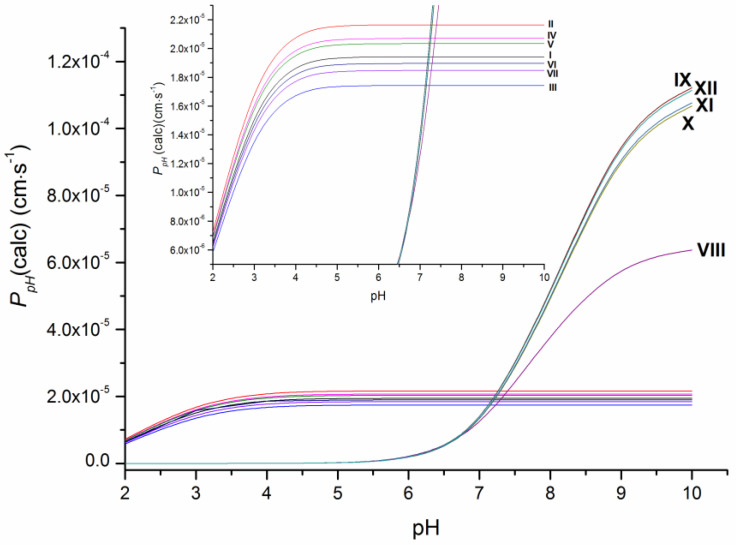
Dependence of the permeability of the studied compounds on pH at 37 °C.

As the ionization constants indicate (Table S2), the investigated compounds have different protolytic properties. Therefore, substances VIII–XII (pK_a_ = 7.8–8.1) containing the piperazine ring are more basic in their character and largely (approximately 75%) ionized, whereas I–VII have only uncharged molecules at pH 7.4. It means that the permeability of the neutral forms of VIII–XII is expected to be higher than the apparent permeability and, thus, it was calculated in this study as P_0_ (Table 1) using the Henderson–Hasselbalch equation based on pK_a_ values (Table S2). Additionally, the calculated permeability at different pH values at 37 °C (P_pH_(calc)) is illustrated in Figure 3. As it shows, the pronounced decrease in the permeability coefficients with the buffer acidity growth is observed for compounds VIII–XII due to the bigger portion of charged cationic species, whereas the permeability coefficients P_pH_(calc) for compounds I–VII became only slightly lower at pH < 4. The revealed tendency seems to be of use for permeability evaluation in media with different pH values.

To summarize the obtained information and to identify the tendencies in the permeability variations, we tried to find correlations between the permeability and the properties of the studied compounds that can be calculated based on the structure or are presented in the literature.

### 2.2. Permeability Correlations

**Figure 4:**
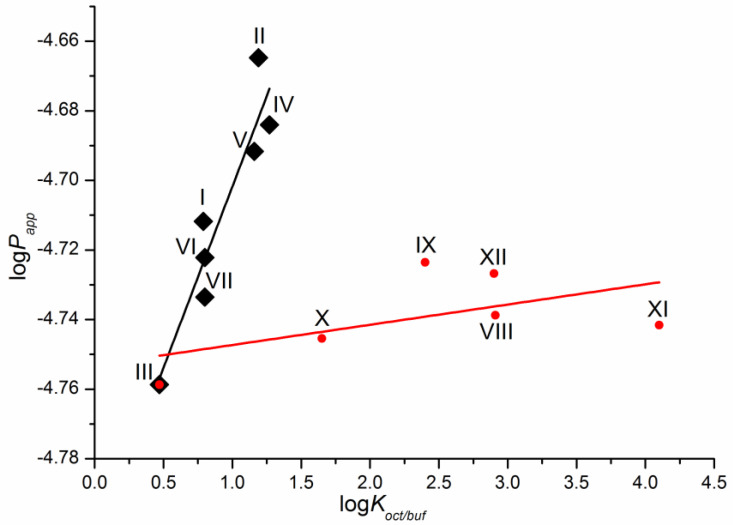
Logarithmic dependence of the experimental permeability coefficient (P_app_) on the distribution coefficient in the 1-octanol/buffer pH 7.4 system (K_oct/buf_).

As a first step, we tried to correlate the permeability of the compounds with the solubility in 1-octanol as a model of the lipophilic medium of the biological membranes and buffer pH 7.4 mimicking the blood plasma fluid taken from the literature [16]. Unfortunately, we did not find any relationships. The dependence of the permeability coefficients on the well-known descriptor—distribution coefficient—is illustrated in Figure 4. The distribution coefficient (logK_oct/buf_) (Table 1) refers to the 1-octanol/buffer pH 7.4 system and was measured experimentally in the literature [16]. Importantly, for compounds VIII–XII, there are both cationic and uncharged particles in the distribution system. Figure 4 clearly shows that the compounds are divided into two groups corresponding to the two dependences obtained. Interestingly, compound III belongs to both dependences. As a result, two correlation equations were derived.

Compounds I–VII

logP_app_ = (−4.81 ± 0.02) + (0.10 ± 0.02) × logK_oct/buf_ (1)

R = 0.9402; F = 38.1; n = 7

Compounds III, VIII–XII

logP_app_ = (−4.75 ± 0.01) + (0.0058 ± 0.0043) × logK_oct/buf_ (2)

R = 0.5612; F = 1.8; n = 6

**Figure 5:**
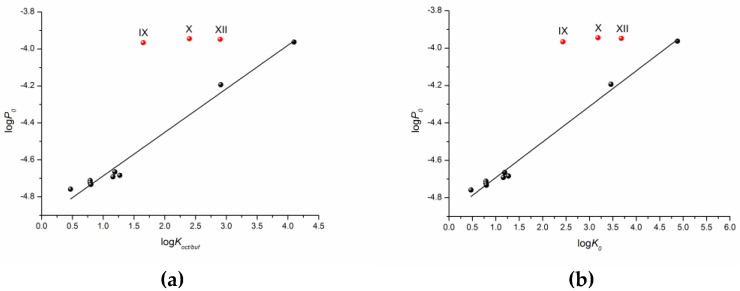
Logarithmic dependences of the calculated permeability coefficient (P_0_) on the experimental distribution coefficient in the 1-octanol/buffer pH 7.4 system (K_oct/buf_) (**a**) and calculated partition coefficient (K_0_) (**b**).

One can see that there is a reliable correlation only for the first group of substances. Bearing in mind the different protolitic properties, we tried to correlate the permeability for the uncharged species (logP_0_) (Table 1) with the distribution coefficient (logK_oct/buf_) (Figure 5a). In addition to this, we calculated the partition coefficients for the neutral particles (logK_0_) by the Henderson–Hasselbalch equation (Table S2) and plotted the logP_0_—logK_0_ dependence (Figure 5b). Figure 5 demonstrates a similar tendency in the logP_0_—logK_oct/buf_ and logP_0_—logK_0_ relationships. However, the correlation coefficient is slightly higher for the latter dependence. It is worth noting that the permeability of three compounds (X–XII) does not satisfy the correlation condition. The following equations were derived:

logP_0_ = (−4.92 ± 0.02) + (0.24 ± 0.01) × logK_oct/buf_ R = 0.9911; F =3 88.2; n = 9(3)

logP_0_ = (−4.88 ± 0.01) + (0.19 ± 0.01) × logK_0_ R = 0.9957; F = 813.5; n = 9(4)

According to the correlation coefficients and especially the Fisher criterion, the correlation taking logK_0_ as the independent variable is somewhat more reliable.

Polarizability (α) is a well-known physicochemical descriptor. Being responsible for the main intermolecular interactions, including the nonspecific (van der Waals) forces, it is often used to predict solubility, permeability, bioactivity and bioavailabilty of drug substances [17,18]. Therefore, as the next step, we tried to determine the interplay between the experimental permeability and polarizability calculated from the structures of the substances (Table S2). The result was not obvious. As many as four compounds (I, III, VI and VII) did not satisfy the linear dependence, although the fitting parameters without these points were rather high: R = 0.9901, F = 299.4, n = 8. The plot and the equation of the correlation are given in the SI file as Figure S1. Taking into account that the compounds excluded from the correlation have different linker fragments and substituents, the mutual influence of the fragments and atoms in the molecule seems to be a possible cause of this phenomenon. Obviously, increasing the number of structural derivatives under study can improve the situation.

**Figure 6:**
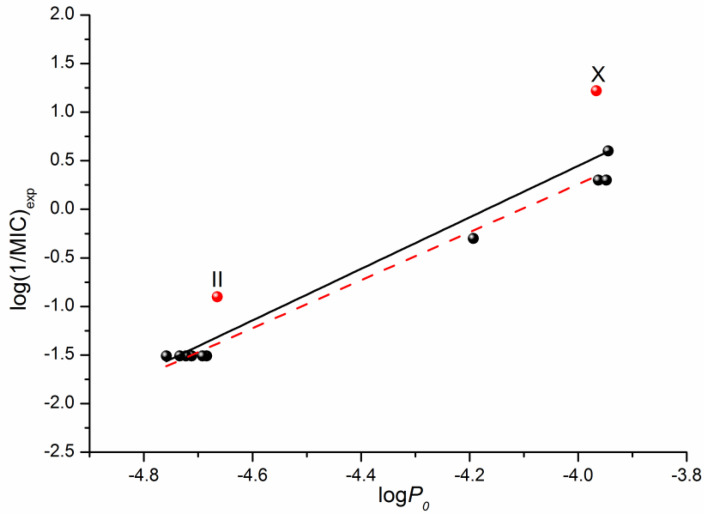
Logarithmic dependences of the calculated permeability coefficient (P_0_) on the antifungal activity log(1/MIC) taken from [16]. The solid black line illustrates the correlation based on all 12 points, whereas the dashed red one on ten points (except II and X).

In addition to the QSPR approach used to predict the properties of drugs and drug-like compounds, there is another approach—the structure–activity relationship (QSAR)—that is aimed to make prognostic models for biological activity based on the structure of a compound and a number of descriptors. Information about the antifungal activity of the considered fluconazole derivatives can be found in the literature [16]. Using the data on the microbiological activity against the strains of the C. parapsilosis ATCC 22019 pathogenic fungi, we tried to correlate the minimum inhibitory concentration (MIC) with the permeability through the PermeaPad barrier measured by us in this study. The respective correlation is illustrated in Figure 6. The following equation was derived through linear fitting:

Log (1/MIC)_exp_ = (11.05 ± 1.07) + (2.65 ± 0.24) × logP_0_ R = 0.9608; F = 120.0; n = 12(5)

Excluding compounds II and X from the correlation that gives Equation (6) improves the correlation coefficient and the Fisher criterion:

log(1/MIC)_exp_ = (11.05 ± 1.07) + (2.65 ± 0.24) × logP_0_ R = 0.9946; F = 739.7; n = 10(6)

As the obtained permeability–activity correlations show, we can predict the in vivo biological activity of the studied class of derivatives by measuring the in vitro permeability through the lipophilic PermeaPad barrier and calculating the permeability for the neutral species using the Henderson–Hasselbalch equation.

**Figure 7:**
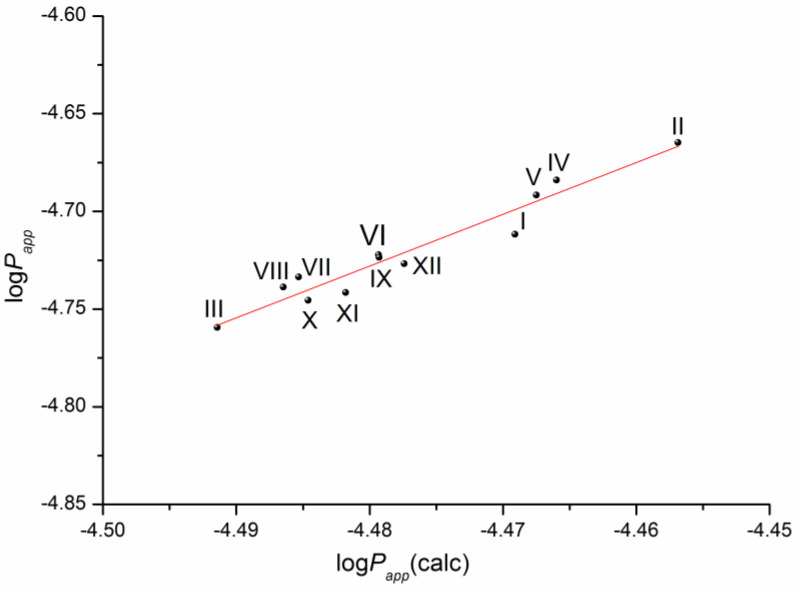
Plot of the dependence between the experimental (P_app_) and calculated (P_app_(calc)) permeability values (Equation (14)) of the selected compounds in a logarithmic scale.

The solubility–diffusion theory previously used in our studies [14,16] was applied to calculate the permeability coefficient by Equations (13)–(15), taking into account the heterogeneous structure of the lipophilic membrane. To this end, the permeability of the lipid and aqueous layers of the membrane was simulated using the diffusion coefficients of the compounds in 1-octanol and water, respectively, as well as the distribution coefficient in the 1-octanol/buffer pH 7.4 system. The plot of the correlation is presented in Figure 7.

The respective equation expressing the dependence between the experimental and the calculated values of the permeability coefficients for the studied compounds is the following:

log(P_app_) = (7.14 ± 0.93) + (2.65 ± 0.21) × log(P_app_)(calc) R= 0.9703; F = 161.0; n = 12(7)

According to the correlation coefficient, the correlation is reliable and can be used to predict the permeability of this class of derivatives using the distribution coefficient in the 1-octanol/buffer pH 7.4 system and the diffusion coefficients of the substances calculated from the structure. 

Not surprisingly, the plots of the log(P_app_)—(P_oct_(calc)) and log(P_app_)—(P_ABL_(calc)) dependences are similar to those of the (log(P_app_)—logK_oct/buf_) and (log(P_app_)—α) dependences, respectively. The plots with the correlation equations and parameters (R and F) are given in the SI file (Figure S2). Evidently, the correlation parameters are better if the diffusion coefficients are taken into account in the calculations. 

## 3. Materials and Methods

### 3.1. Materials and Reagents

The objects of the present study are the following derivatives of the antifungal drug fluconazole (hybrids of thiazolo[4,5-d]pyrimidines with (1H-1,2,4)triazoles synthesized in the Gause Institute of New Antibiotics): N-[2-(2,4-difluorophenyl)-2-hydroxy-3-(1,2,4-triazol-1-yl)propyl]-3-(p-tolyl)-2-thioxo-thiazolo[4,5-d]pyrimidin-7-one (I), N-[2-(2,4-difluorophenyl)-2-hydroxy-3-(1,2,4-triazol-1-yl)propyl]-3-(2-methoxyphenyl)-2-thioxo-thiazolo[4,5-d]pyrimidin-7-one (II), N-[2-(2,4-difluorophenyl)-2-hydroxy-3-(1,2,4-triazol-1-yl)propyl]-3-(4-fluorophenyl)-2-thioxo-thiazolo[4,5-d]pyrimidin-7-one (III), N-[2-(2,4-difluorophenyl)-2-hydroxy-3-(1,2,4-triazol-1-yl)propyl]-2-[7-oxo-3-(p-tolyl)-2-thioxo-thiazolo[4,5-d]pyrimidin-6-yl]acetamide (IV), N-[2-(2,4-difluorophenyl)-2-hydroxy-3-(1,2,4-triazol-1-yl)propyl]-2-[3-(4-chlorophenyl)-7-oxo-2-thioxo-thiazolo[4,5-d]pyrimidin-6-yl]- acetamide (V), N-[2-(2,4-difluorophenyl)-2-hydroxy-3-(1,2,4-triazol-1-yl)propyl]-2-[3-(4-fluorophenyl)-7-oxo-2-thioxo-thiazolo[4,5-d]pyrimidin-6-yl]acetamide (VI), N-[2-(2,4-difluorophenyl)-2-hydroxy-3-(1,2,4-triazol-1-yl)propyl]-2-[3-(2-methoxyphenyl)-7-oxo-2-thioxo-thiazolo[4,5-d]pyrimidin-6-yl]acetamide (VII), N-[3-[4-[2-(2,4-difluorophenyl)-2-hydroxy-3-(1,2,4-triazol-1-yl)propyl]piperazin-1-yl]-2-hydroxy-propyl]-3-(2-methoxyphenyl)-2-methyl-2-thioxo-thiazolo[4,5-d]pyrimidin-7-one (VIII), N-[3-[4-[2-(2,4-difluorophenyl)-2-hydroxy-3-(1,2,4-triazol-1-yl)propyl]piperazin-1-yl]propyl]-3-(4-fluorophenyl)-2-methyl-2-thioxo-thiazolo[4,5-d]pyrimidin-7-one (IX), N-[3-[4-[2-(2,4-difluorophenyl)-2-hydroxy-3-(1,2,4-triazol-1-yl)propyl]piperazin-1-yl]propyl]-3-(4-chlorophenyl)-2-methyl-2-thioxo-thiazolo[4,5-d]pyrimidin-7-one (X), N-[3-[4-[2-(2,4-difluorophenyl)-2-hydroxy-3-(1,2,4-triazol-1-yl)propyl]piperazin-1-yl]propyl]-3-(p-tolyl)-2-thioxo-thiazolo[4,5-d]pyrimidin-7-one (XI), N-[2-[4-[2-(2,4-difluorophenyl)-2-hydroxy-3-(1,2,4-triazol-1-yl)propyl]piperazin-1-yl]ethyl]-2-methyl-3-(p-tolyl)-2-thioxo-thiazolo[4,5-d]pyrimidin-7-one (XII). The synthesis procedure and ^1^H NMR spectra of the compounds are described in detail in the study by Blokhina et al. [16].

Bidistilled water (with electrical conductivity of 2.1 μS cm^−1^) was used to prepare the buffer solution pH 7.4 (I = 0.26 mol·dm^−3^) from KH_2_PO_4_ (9.1 g in 1 L) and Na_2_HPO_4_·12H_2_O (23.6 g in 1 L) salts. The reagents for the buffer preparation were received from Merk: potassium dihydrogen phosphate (purity 99%) and disodium hydrogen phosphate dodecahydrate (purity 99%).

### 3.2. Membrane Permeability Assay

A vertical-type Franz diffusion cell (PermeGear, Inc., Hellertown, PA, USA) was used to measure the permeability of the studied compounds. The biomimetic lipophilic PermeaPad barrier (PHABIOC, Germany, www.permeapad.com, accessed on 20 May 2021) fully described by di Cagno et al. [18] was employed in the present study to simulate the morphology and properties of the biologic membranes. This membrane was chosen because of its advantageous properties and user friendliness. PermeaPad belongs to cell-free permeation systems. It is composed of two cellulose membranes with a thin layer of phosphatidylcholine (S-100) between them. Contacting with water, the phospholipids generate a tightly packed layer consisting of stacks of bilayers with intercalating water layers, thus, mimicking the biologic membrane structure [19]. The barrier is mechanically flexible, withstands a wide spectrum of pHs (pH 1–pH 9), does not require any pretreatment and can be used as received [18]. 

Firstly, the donor chamber was filled with the donor solution of the tested compound in buffer pH 7.4, then the PermeaPad barrier was mounted between the donor and receptor parts of the diffusion cell (0.785 cm^2^ effective surface area) carefully avoiding the air bubbles (which can minimize the active area of the membrane, thus, slowing the permeation). Then, the receptor chamber was placed onto the membrane, fastened with a clip and filled with pure buffer pH 7.4 to simulate the compound transition to the blood flow. The volumes of the donor and receptor solutions were 7.0 and 1.0 mL, respectively. The temperature was maintained at 37.0 ± 0.1 °C throughout the experiment. The donor solution was stirred with a magnetic stirrer bar (500 rpm). Samples of 0.5 mL were taken out of the receptor solution every 30 min and replaced with an equal amount of the respective fresh buffer. The experiment lasted 5 h. The sample solution concentrations were measured spectrophotometrically (Spectramax 190; Molecular Devices, Molecular Devices Corporation, San Jose, CA, USA) in 96-well UV black plates (Costar) at λ = 270 nm. The amount of the permeated drug (dQ/A) was plotted versus time (t), taking into account the effective surface area of the membrane. The slope of the permeation plot produced the flux (J):

(8) $$J=\frac{\mathrm{dQ}}{A\cdot \mathrm{dt}}$$

The apparent permeability coefficient (P_app_) was calculated by normalizing the slope (J) by the compound concentration in the donor solution (C_0_) by the equation:

(9) $$P_{\mathrm{app}}=\frac{J}{C_{0}}$$

The average value of P_app_ was taken from at least 3 replicated experiments.

### 3.3. Calculation Procedure within the Solubility–Diffusion Theory

#### 3.3.1. Calculation of the Diffusion Coefficients in Water and 1-octanol

The Stokes−Einstein equation was applied to calculate the diffusion coefficients in water and 1-octanol:

(10) $$D=\frac{\mathrm{kT}}{6\pi r\eta }$$

where k is the Boltzmann constant, T is the absolute temperature, η is the dynamic viscosity of the solvent and r is the solute hydrodynamic radius.

The van der Waals volumes of the molecules were calculated by the group contribution approach [20]. Since the particles or molecules were assumed to be spherical, the solute hydrodynamic radii were determined from the van der Waals volumes as follows [21]:

(11) $$r={\left(\frac{3V_{\mathrm{vdw}}}{4\pi }\right)}^{1/3}$$

where V_vdw_ is the van der Waals volume of the permeating molecule.

#### 3.3.2. Calculation of the Permeability Coefficients

The well-known solubility–diffusion theory was applied to calculate the permeability coefficient, taking into account the oil-like nature of the biological membrane [22]. Expressed mathematically, the transition of the molecule through the lipophilic layer of the membrane is ruled by its ability to partition into the lipid phase and diffusion coefficient within the phase and can be expressed as:

(12) $$P_{\mathrm{app}}=\frac{K\cdot D}{h}$$

where K, D and h are the lipid/water partition coefficient, the solute diffusion coefficient in the lipid phase and the thickness of the lipid membrane, respectively.

Taking into account that 1-octanol, an amphiphilic solvent, serves as the lipophilic medium model, the coefficient of permeability through the 1-octanol layer is assumed to simulate the lipophilic membrane. Thus, it can be calculated by the equation:

(13) $$P_{\mathrm{oct}}(\mathrm{calc})=\frac{D_{\mathrm{oct}}\cdot K_{\mathrm{oct}/\mathrm{buf}}}{h_{\mathrm{oct}}}$$

where D_oct_ is the diffusion coefficient of the investigated substance in 1-octanol, K_oct/buf_ is the distribution coefficient in the 1-octanol/buffer pH 7.4 system and h_oct_ is the 1-octanol layer thickness. 

The value of h_oct_ =100 μm was taken as the thickness of the analogous artificial membrane [23]. The natural membrane is known to be inhomogeneous. Since it contains phospholipids and other amphiphilic molecules, a bilayer structure is formed when the polar “head” groups of the lipids are turned to the external border of the aqueous phase and the lipophilic “tails” are extended toward the center of the bilayer. The ability of the membrane surface to form hydrogen bonds with water was stated in [24]. This feature lowers (by more than one order of magnitude) the mobility of the water molecules directly adsorbed on the surface promoting the formation of an Aqueous Boundary Layer (ABL). The thickness of this layer in the human intestinal tract ranges from 10 to 100 μm [25]. The total resistance of the membrane consists of a sum of the resistances of the individual layers (lipid and water) and can be expressed by the equation:

(14) $$\frac{1}{P_{\mathrm{app}}}=\frac{1}{P_{\mathrm{ABL}}}+\frac{1}{P_{\mathrm{Lip}}}$$

where P_app_ is the total coefficient of permeability through the water and lipid layers and P_ABL_ and P_Lip_ are the coefficients of permeability through the water and lipid layers, respectively. 

In the case of the in vitro permeability measurement, the resistance of the water layer at the receptor side can be assumed to be negligible due to the rapid removal of the drug from the membrane surface in the diluted receptor solution [25]. It means that passive diffusion through the phospholipid membrane can be considered an additive process of the molecule passage through the structured water barrier and the lipid layer of the cell membrane (Equation (15)). As has been said before, the lipid layer permeability coefficient is calculated (Equation (13)) taking 1-octanol as the model of this layer. Similarly, the water layer permeability coefficient can be calculated by the equation:

(15) $$P_{\mathrm{ABL}}(\mathrm{calc})=\frac{D_{w}}{h_{\mathrm{ABL}}}$$

where D_w_ is the diffusion coefficient of the investigated substance in H_2_O; h_ABL_ is the water layer thickness; and the partition coefficient of the substance in water is equal to 1.

The thickness of the water layer cannot be determined precisely [26] and was assumed to be 2000 μm as the maximal possible value for in vitro conditions [19]. The thickness of the 1-octanol and water layers taken as 100 μm and 2000 μm, respectively, was successfully applied in our previous study [9], where a reliable correlation between the experimental and calculated permeability was obtained in the framework of the diffusion theory.

## 4. Conclusions

The apparent permeability coefficients of new fluconazole derivatives with triazole and thiazolo-pyrimidine moieties connected by a variety of linker bridges through the lipophilic PermeaPad barrier were determined for the first time. Taking into account the significance of predicting the permeability of the newly synthesized drug substances, we found the correlations of their permeability with the physicochemical properties and activity. The permeability coefficients of the compounds within the studied class were shown to depend both on the structure of the linker fragment and on the substituent in the phenyl ring of the thiazolo-pyrimidine moiety. The presence of the acceptor nitrogen atom in the linker appeared to be a negative factor leading to a permeability reduction. Using the dependence of the permeability coefficients on the distribution coefficients of the substances in the 1-octanol/buffer pH 7.4 system, we only found a reliable correlation (R = 0.9402) for compounds (I–VII), which were unionized at the experimental pH 7.4. Recalculating the permeability coefficients of compounds (VIII-XII) to the uncharged particles, we were able to improve the correlation by bringing it to R = 0.9911 (compounds IX, X, XII were excluded). We found a reliable correlation (R = 0.9608) of the permeability coefficients for the uncharged species of the studied derivatives on the antifungal activity, which proved it was possible to predict the in vivo biological activity within the studied class by measuring the in vitro permeability. We also derived an important correlation based on the data on the whole series of the studied compounds within the framework of the solubility–diffusion approach (R = 0.9703) using the calculated diffusion coefficients in 1-octanol and water, as well as the experimental distribution coefficients in the 1-octanol/buffer pH 7.4 system. There are but a few works where this approach is used, so we hope that our study will be of interest to scientists dealing with permeability investigation and prediction. Moreover, the obtained experimental data can enrich the in vitro permeability databases of drug and drug-like compounds.

## Figures and Tables

**Table 1:** Van der Waals volumes (V_vdw_), diffusion coefficients in water (D_w_) and 1-octanol (D_oct_), apparent distribution coefficient in the 1-octanol/buffer pH 7.4 system (K_oct/buf_), apparent permeability coefficients (P_app_), permeability coefficients calculated for the neutral species (P_0_), calculated coefficients of permeability through the water layer (P_ABL_(calc)), 1-octanol layer (P_oct_(calc)) and calculated total coefficient of permeability through both layers (P_app_(calc)) for the studied compounds.

№	V_vdw_ (Å^3^) ^*a*^	D_w_∙10^10^ (m^2^∙s^−1^) ^*b*^	D_oct_∙10^11^ (m^2^∙s^−1^) ^*b*^	logK_oct/buf_ *^c^*	P_app_⋅10^5^ (cm∙s^−1^) *^c^*	P_0_ ⋅10^5^ (cm∙s^−1^) *^d^*	P_ABL_(calc)⋅10^5^ (cm∙s^−1^) *^e^*	P_oct_(calc) (cm∙s^−1^) *^f^*	P_app_(calc)⋅ 10^5^ (cm∙s^−1^) *^g^*
**I**	414.24	7.21	9.55	0.79	1.942 ± 0.078	1.942	3.60	5.89 × 10^−4^	3.40
**II**	423.22	7.16	9.48	0.47	2.164 ± 0.059	2.164	3.58	1.47 × 10^−3^	3.49
**III**	402.61	7.28	9.64	1.19	1.743 ± 0.047	1.743	3.64	2.85 × 10^−4^	3.23
**IV**	456.19	6.98	9.25	1.27	2.070 ± 0.074	2.070	3.49	1.72 × 10^−3^	3.42
**V**	453.16	6.99	9.27	0.79	2.034 ± 0.066	2.034	3.50	1.34 × 10^−3^	3.41
**VI**	444.56	7.04	9.33	1.16	1.896 ± 0.058	1.896	3.52	5.75 × 10^−4^	3.32
**VII**	465.17	6.93	9.19	0.80	1.847 ± 0.033	1.847	3.47	5.80 × 10^−4^	3.27
**VIII**	557.37	6.53	8.65	2.91	1.825 ± 0.058	6.409	3.26	7.03 × 10^−2^	3.26
**IX**	528.71	6.64	8.81	2.40	1.890 ± 0.064	11.36	3.32	2.21 × 10^−2^	3.32
**X**	537.31	6.61	8.76	1.65	1.797 ± 0.047	10.80	3.30	3.91 × 10^−3^	3.28
**XI**	540.34	6.60	8.74	4.10	1.813 ± 0.045	10.90	3.30	1.10	3.30
**XII**	523.53	6.67	8.83	2.90	1.876 ± 0.042	11.28	3.33	7.02 × 10^−2^	3.33

*^a^* calculated from the structure; *^b^* calculated by Equation (10); *^c^* taken from literature [16]; *^d^* calculated by the Henderson–Hasselbalch equation; *^e^* calculated by Equation (15); *^f^* calculated by Equation (13); *^g^* calculated by Equation (14).

## Data Availability

The results obtained in all the experiments performed are shown in the manuscript and SI; the raw data will be provided upon request.

## Supplementary Materials

The following supporting information can be downloaded at: https://www.mdpi.com/article/10.3390/molecules28010389/s1. Figure S1: Logarithmic dependences of the apparent permeability coefficient (logP_app_) on polarizability (α). The correlation equation: logP_app_ = (−4.39 ± 0.02) − (0.0053 ± 0.0003)·α, R = 0.9901, F = 299.4, n = 8; Figure S2: Logarithmic dependences of the experimental apparent permeability coefficient (P_app_) on the calculated permeability coefficient through the 1-octanol layer P_oct_ (a) and water layer P_ABL_ (b); Table S1: Donor solution concentrations (C), steady penetration rate (J) for the studied compounds at pH 7.4 and 37 °C (for compounds numbering see Figure 1); Table S2: Physicochemical parameters: polarizability (α), pK_a_, distribution coefficients for the uncharged particles (K_0_), and minimum inhibitory concentrations for strains of pathogenic fungi C. parapsilosis ATCC 22019 (MIC) for the studied compounds.

## Sample Availability

Samples of the compounds (I–XII) are available from the authors.
