# Supplementary material for: Permeability of New Antifungal Fluconazole Derivatives through a Lipophilic Membrane: Experiment and Modeling
Source: Molecules. 2023 Jan 2;28(1):389. doi: 10.3390/molecules28010389 (PMC9823331; doi:10.3390/molecules28010389)
Supplement: Supplementary file 1 [file molecules-28-00389-s001.zip › molecules-2101630-supplementary.pdf]

Supplementary Material

Permeability of new antifungal fluconazole derivatives through a lipophilic membrane: Experiment and modeling

Tatyana V. Volkova*, German L. Perlovich

G.A. Krestov Institute of Solution Chemistry RAS, 153045 Ivanovo, Russia
* Correspondence: 1 Akademicheskaya str., 153045 Ivanovo, Russian Federation, vtv@isc-ras.ru

Table of Contents

Figure S1	Logarithmic dependences of the apparent permeability coefficient ($logP_{app}$) on the polarizability (α). The correlation equation: $logP_{app}$ =(-4.39±0.02)-(0.0053±0.0003)·α, R=0.9901, F=299.4, n=8.	Page 2
Figure S2	Logarithmic dependences of the experimental apparent permeability coefficient (P_{app}) on the calculated permeability coefficient through the 1-octanol layer P_{oct} (a) and water layer P_{ABL} (*b*).	Page 2
Table S1	Donor solution concentrations (C), steady penetration rate (J) for the studied compounds at pH 7.4 and 37 °C (for compounds numbering see Figure 1).	Page 3
Table S2	Physicochemical parameters: polarizability (α), pK_a, and minimum inhibitory concentrations for strains of pathogenic fungi C. parapsilosis ATCC 22019 (MIC) for the studied compounds.	Page 3

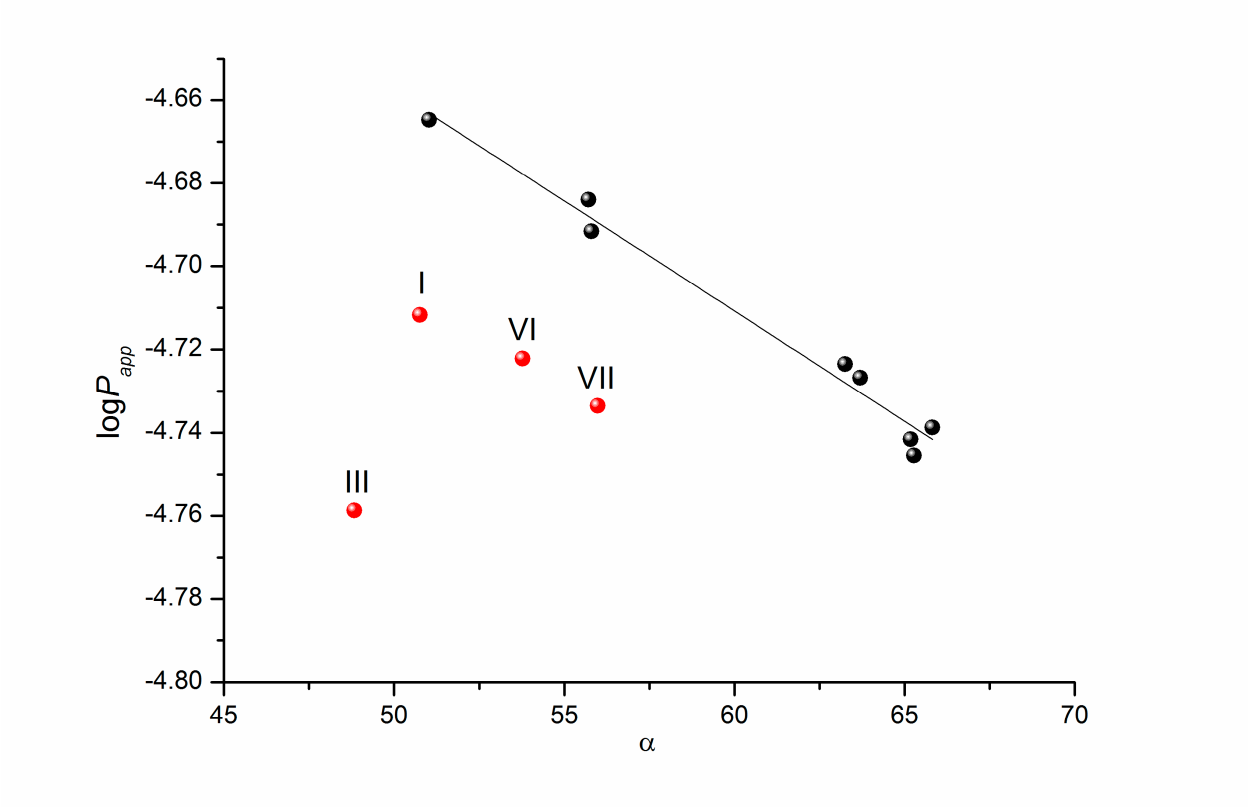

Figure S1. Logarithmic dependences of the apparent permeability coefficient (logP_{app}) on the polarizability (α). The correlation equation: logP_{app} =(-4.39±0.02)-(0.0053±0.0003)·α, R=0.9901, F=299.4, n=8.

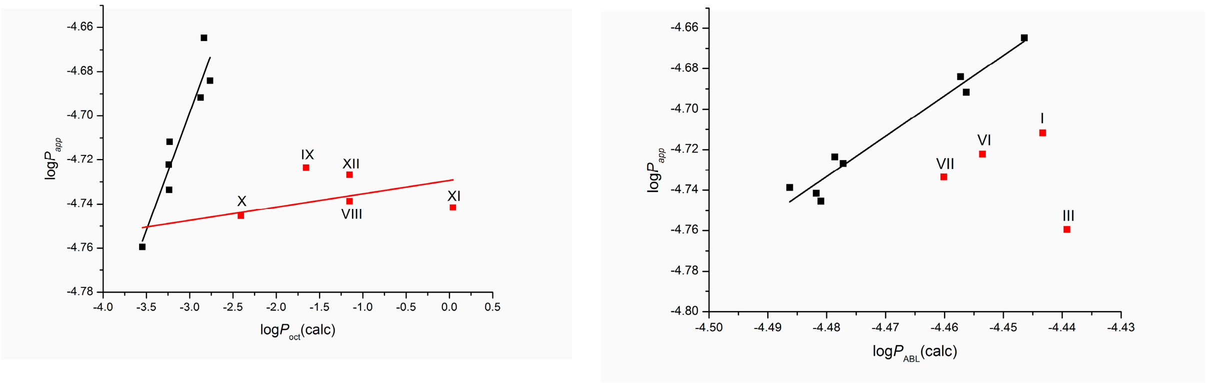

(a)
(I-VII)
$\log P_{app}=(-4.38\pm0.06)+(0.11\pm0.016)\cdot\log P_{oct}(calc)$
R=0.9464; F=42.9; n=7
(III, VIII-XII)
$\log P_{app}=(-4.73\pm0.01)+(0.006\pm0.004)\cdot\log P_{oct}(calc)$
R=0.559; F=1.9; n=6

(b)
(II, IV-VI, VIII-XII)
$\log P_{app}=(4.19\pm0.77)+(1.99\pm0.17)\cdot\log P_{ABL}(calc)$
R=0.9783; F=133.53; n=8

Figure S2. Logarithmic dependences of the experimental apparent permeability coefficient (P_{app}) on the calculated permeability coefficient through the 1-octanol layer (P_{oct}(calc)) (a) and water layer (P_{ABL}(calc)) (*b*).

Table S1. Donor solution concentrations (C), steady penetration rate (J) for the studied compounds at pH 7.4 and 37 °C (for compounds numbering see Fig. 1).

Compound	C (M)	J (μM·cm^{-2}·s^{-1})	Compound	C (M)	J (μM·cm^{-2}·s^{-1})
**I**	$7.010 \cdot 10^{-5}$	$1.361 \cdot 10^{-6}$	**VII**	$7.643 \cdot 10^{-5}$	$1.412 \cdot 10^{-6}$
**II**	$1.825 \cdot 10^{-4}$	$3.950 \cdot 10^{-6}$	**VIII**	$2.530 \cdot 10^{-4}$	$4.618 \cdot 10^{-6}$
**III**	$6.120 \cdot 10^{-5}$	$1.067 \cdot 10^{-6}$	**IX**	$8.409 \cdot 10^{-5}$	$1.589 \cdot 10^{-6}$
**IV**	$1.610 \cdot 10^{-4}$	$3.332 \cdot 10^{-6}$	**X**	$6.673 \cdot 10^{-5}$	$1.199 \cdot 10^{-6}$
**V**	$1.110 \cdot 10^{-4}$	$2.258 \cdot 10^{-6}$	**XI**	$1.200 \cdot 10^{-4}$	$2.176 \cdot 10^{-6}$
**VI**	$6.318 \cdot 10^{-5}$	$1.198 \cdot 10^{-6}$	**XII**	$1.658 \cdot 10^{-5}$	$3.110 \cdot 10^{-7}$

Table S2. Physicochemical parameters: polarizability (α), pK_a, distribution coefficients for the uncharged particles (K_0), and minimum inhibitory concentrations for strains of pathogenic fungi C. parapsilosis ATCC 22019 (MIC) for the studied compounds.

Compound number	M (g·mol^{-1})	α (A^3) ^{a}	pK_a^{b}	logK_0^{c}	log(1/MIC) ^{d}
**I**	512.55	414.24	2.3	-4.71	-1.51
**II**	528.55	423.22	2.3	-4.66	-0.90
**III**	516.52	402.61	2.3	-4.76	-1.51
**IV**	569.61	456.19	2.3	-4.68	-1.51
**V**	590.02	453.16	2.3	-4.69	-1.51
**VI**	573.09	444.56	2.3	-4.72	-1.51
**VII**	585.60	465.17	2.3	-4.73	-1.51
**VIII**	687.80	557.37	7.8	-4.19	-0.30
**IX**	657.75	528.71	8.1	-3.94	1.22
**X**	674.21	537.31	8.1	-3.97	0.30
**XI**	638.75	540.34	8.1	-3.96	0.6
**XII**	639.21	523.53	8.1	-3.95	0.30

^{a}calculated from the structure; ^{b}calculated by pDISOL-X program; ^{c}calculated by Henderson–Hasselbalch equation; ^{d}taken from literature [16]
